# Accumulation of Soil Carbon and Phosphorus Contents of a Rehabilitated Forest

**DOI:** 10.1100/tsw.2010.196

**Published:** 2010-10-12

**Authors:** Osumanu Haruna Ahmed, Nur Aainaa Hasbullah, Nik Muhamad Ab Majid

**Affiliations:** ^1^ Department of Crop Science, Faculty of Agriculture and Food Sciences, Universiti Putra Malaysia, Bintulu Sarawak Campus, Bintulu, Sarawak, Malaysia; ^2^ Department of Forest Management, Faculty of Forestry, Universiti Putra Malaysia, Serdang, Selangor, Malaysia

**Keywords:** soil carbon, soil phosphorus, tropical forests, forest rehabilitation, degraded soils

## Abstract

The world's tropical rainforests are decreasing at an alarming rate as they are converted to agricultural land, pasture, and plantations. Decreasing tropical forests affect global warming. As a result, afforestation progams have been suggested to mitigate this problem. The objective of this study was to determine the carbon and phosphorus accumulation of a rehabilitated forest of different ages. The size of the study area was 47.5 ha. Soil samples were collected from the 0-, 6-, 12-, and 17-year-old rehabilitated forest. Twenty samples were taken randomly with a soil auger at depths of 0–20 and 20–40 cm. The procedures outlined in the Materials and Methods section were used to analyze the soil samples for pH, total C, organic matter, total P, C/P ratio, yield of humic acid (HA), and cation exchange capacity (CEC). The soil pH decreased significantly with increasing age of forest rehabilitation regardless of depth. Age did not affect CEC of the rehabilitated forest. Soil organic matter (SOM), total C, and total P contents increased with age. However, C/P ratio decreased with time at 0–20 cm. Accumulation of HA with time and soil depth was not consistent. The rehabilitated forest has shown signs of being a C and P sink.

## INTRODUCTION

Malaysia is one of the mega biodiversity countries in the world. The tropical rainforest of Malaysia houses over 8,000 species of flowering plants, of which 2,500 are tree species. The natural forest plays significant roles in protecting the water supply, thus ensuring environmental stability and maintenance of climatic patterns of the country. As a developing country, Malaysia has to strike a balance between development and conservation of the tropical forest. The deforestation rate in Peninsular Malaysia has been estimated to be 37,690 ha annually from 1979 to 1992[1]. The forest is decreasing at a rate of 16.9 million ha/year because of its conversion to agricultural land, pasture, and plantations. Decreasing tropical forests affect global warming because deforestation accelerates greenhouse gas emissions and decreases the accumulation of carbon dioxide ($CO_2$) through photosynthesis of tropical trees[2].

In order to reverse deforestation, a rehabilitation program initiated by Universiti Putra Malaysia (UPM) and the Japanese Centre for International Studies in Ecology since 1990 has enabled establishment of indigenous tree species on a degraded land area. This was a remarkable achievement because tropical forests have the largest potential to mitigate climate change among the world's forests through conservation of existing carbon (C) pools (e.g., reduction of impact of logging), expansion of C sinks (e.g., reforestation, agroforestry), and substitution of wood products for fossil fuels[3].

Soils constitute the third largest C pool (2,300 Gt or billion tons), after oceanic (38,000 Gt) and geologic (5,000 Gt) pools. The soil C pool is directly linked with the biotic (600 Gt) and atmospheric (770 Gt) pools. According to Lal[4], an increase in the soil C pool by 1 Gt will reduce the rate of atmospheric enrichment of $CO_2$ by 0.47 ppm. At the moment, little is known about the trend of soil C and phosphorus (P) accumulation in relation to different ages of rehabilitated forests. Many factors, such as soil pH, soil cation exchange capacity (CEC), humus, type of soil, and organic matter, are also related to availability of C, P, and other nutrients that promote soil fertility. Information on C and P accumulation of rehabilitated forests suggests or indicates whether these forests can serve as C and P sinks to mitigate climate change. Thus, the main purpose of this study was to determine C and P accumulation over 20 years of rehabilitating a degraded forest on an acid soil.

## MATERIALS AND METHODS

A study on soil C and P accumulation in a rehabilitated forest was conducted at the JISE-UPM-UNIMAS rehabilitated forest at Universiti Putra Malaysia Bintulu Campus Sarawak, Malaysia. An auger was used to take soil samples from the 0-, 6-, 12-, and 17-year-old rehabilitated forest. Soil sampling for all of the ages of the rehabilitated forest was done at the same time to avoid bias. The soil was Typic Paleudalts. There were four plots for each of the ages of the rehabilitated forest. The size of each plot was 20 × 20 m. A total of 20 soil samples were taken at random depths of 0–20 and 20–40 cm in each plot for each age. Each sample was a bulk of three samples. These soil samples were air dried, crushed manually, and sieved to pass a 2-mm sieve, after which they were transferred into plastic bags and labeled.

The subsequent soil chemical analyses below were carried out at the same time, i.e., they were not analyzed on an age-by-age basis (not separately) to avoid bias. The pH was determined in a ratio 1:2.5 of soil: distilled water suspension and/or 1 M KCl using a glass electrode[5]. The ammonium acetate (leaching) method was used to determine soil CEC[6]. Soil organic matter (SOM) and total organic carbon (TOC) were determined by the method of Chefetz et al.[7]. A 5-g sample of soil was weighed into a crucible using a digital balance. The weight of the soil and crucible was determined. Afterwards, they were placed in a muffle furnace and ashed. The soil samples were initially ashed at 300°C for 1 h, after which the temperature was raised to 750°C for 5 h. Afterwards, the sample was weighed upon cooling in a desiccator and simple mathematics was used to determine SOM. The calculated organic matter multiplied by 0.58 provides soil TOC. Total P was determined using the aqua regia and the blue methods. A 2-g sample of soil was placed in a 250-mL volumetric flask. A 20-mL portion of the aqua regia solution (Mixture of HCl and $HNO_3$ in a ratio of 3:1) was added. The sample was heated under reflux for 30 min until the solution was clear. The solution was filtered through ashless filter (Whatman filter paper No. 2) into a volumetric flask of 50 mL and diluted to volume with distilled water. The total P was determined using a UV-VIS spectrophotometer.

Independent t-test was used to detect significant difference for SOM, TOC, total P, CEC, and pH at the different soil depths of the rehabilitated forest. Analysis of variance (ANOVA) was used to detect significance of SOM, TOC, total P, CEC, and pH of the different ages of the rehabilitated forest while Duncan's new multiple range test was used for mean comparison. The Statistical Analysis System (SAS) version 9.1 was used for the statistical analysis.

## RESULTS AND DISCUSSION

The soil texture at 0–20 and 20–40 cm for all the ages of the rehabilitated forest was loamy sand. Soil pH in water was not affected by the depth of the rehabilitated forest, but the contrary was true for soil pH in KCl (Table 1). In terms of age, the results of the statistical means comparison (Table 2) were (1) soil pH (1 M KCl) between different ages at 0–20 cm – significant difference; (2) soil pH between different ages at 20–40 cm – significant difference; (3) soil pH (water) between different ages at 0–20 cm – no significant difference; (4) soil pH between 0 and 6 years at 20–40 cm – significant difference; (5) soil pH between 12 and 17 years at 20–40 cm – no significant difference. These pH values in water and 1 M KCl were consistent with those reported by Leng et al.[8]. The authors reported a range of 4.2–4.3 for pH in water and 3.5–3.6 for pH in KCl.

**TABLE 1**
**pH of a Rehabilitated Forest Soil (between Depths)**

| Age of Rehabilitated Forest Soil | pH (Water) | pH (1 M KCl) |
|---|---|---|
| 2008 (0 years old) | | |
| 0–20 cm | $4.430^{a} \pm 0.0532$ | $3.847^{b} \pm 0.0175$ |
| 20–40 cm | $4.399^{a} \pm 0.0317$ | $3.918^{a} \pm 0.0314$ |
| 2002 (6 years old) | | |
| 0–20 cm | $4.524^{b} \pm 0.0219$ | $3.564^{b} \pm 0.0123$ |
| 20–40 cm | $4.629^{a} \pm 0.0152$ | $3.658^{a} \pm 0.0168$ |
| 1996 (12 years old) | | |
| 0–20 cm | $4.402^{a} \pm 0.0964$ | $3.233^{b} \pm 0.0194$ |
| 20–40 cm | $4.439^{a} \pm 0.0430$ | $3.288^{a} \pm 0.0168$ |
| 1991 (17 years old) | | |
| 0–20 cm | $4.537^{a} \pm 0.0342$ | $3.394^{a} \pm 0.0164$ |
| 20–40 cm | $4.551^{a} \pm 0.0305$ | $3.428^{a} \pm 0.0146$ |

*Note*: Means within column with different letters indicate significant difference between depths by independent t-test at $p \leq 0.05$.

**TABLE 2**
**pH of a Rehabilitated Forest Soil (Different Ages)**

| Age of Rehabilitated Forest Soil | Soil pH | |
|---|---|---|
| | Water | 1 M KCl |
| Soil depth (0–20 cm) | | |
| 2008 (0 years old) | $4.430^{a}$ | $3.847^{a}$ |
| 2002 (6 years old) | $4.524^{a}$ | $3.564^{b}$ |
| 1996 (12 years old) | $4.402^{a}$ | $3.233^{d}$ |
| 1991 (17 years old) | $4.537^{a}$ | $3.394^{c}$ |
| Soil depth (20–40 cm) | | |
| 2008 (0 years old) | $4.399^{b}$ | $3.918^{a}$ |
| 2002 (6 years old) | $4.629^{a}$ | $3.658^{b}$ |
| 1996 (12 years old) | $4.439^{b}$ | $3.288^{d}$ |
| 1991 (17 years old) | $4.551^{a}$ | $3.428^{c}$ |

*Note*: Means within column with different letters indicate significant difference in different ages by Duncan test at $p \leq 0.05$.

In terms of depth, the SOM and total C at 0–20 cm of the 6- and 12-year-old rehabilitated forest were higher than those at 20–40 cm (Table 3). In terms of age, the statistical comparison of means (Table 4) revealed the following: (1) SOM of the 6-year-old rehabilitated forest at 0–20 cm compared to the 0-, 12-, and 17-year-old rehabilitated forest – significant difference; (2) SOM of the 0-, 12-, and 17-year-old rehabilitated forest at 0–20 cm – no significant difference; (3) SOM of the 12- and 17-year-old rehabilitated forest at 20–40 cm – significant difference; (4) SOM of the 0- and 6-year-old forest at the same depth – no significant difference; (5) total C of the 0-, 6-, 12-, and 17-year-old rehabilitated forest regardless soil depth – significant difference. The values of SOM and total C reported by Leng et al.[8] in a rehabilitated forest regardless of age were consistent with those of this study except for 0 years old, which was slightly higher. But at 0 years old, the value recorded in this study was lower. The data from Leng et al.[8] on SOM ranged between 5 and 6%, while the total C ranged between 3 and 5%.

**TABLE 3**
**SOM and Total C of a Rehabilitated Forest (between Depths)**

| Age of Rehabilitated Forest Soil | SOM (%) | Total C (%) |
|---|---|---|
| 2008 (0 years old) | | |
| 0–20 cm | $3.485^{a} \pm 0.149$ | $2.022^{a} \pm 0.087$ |
| 20–40 cm | $3.370^{a} \pm 0.544$ | $1.955^{a} \pm 0.316$ |
| 2002 (6 years old) | | |
| 0–20 cm | $5.769^{a} \pm 0.233$ | $3.346^{a} \pm 0.135$ |
| 20–40 cm | $4.175^{b} \pm 0.131$ | $2.422^{b} \pm 0.076$ |
| 1996 (12 years old) | | |
| 0–20 cm | $3.762^{a} \pm 0.161$ | $2.182^{a} \pm 0.093$ |
| 20–40 cm | $2.440^{b} \pm 0.142$ | $1.415^{b} \pm 0.082$ |
| 1991 (17 years old) | | |
| 0–20 cm | $5.355^{a} \pm 0.166$ | $3.106^{a} \pm 0.096$ |
| 20–40 cm | $5.492^{a} \pm 0.613$ | $3.185^{a} \pm 0.342$ |

*Note*: Means within column with different letters indicate significant difference between depths by independent t-test at $p \leq 0.05$.

**TABLE 4**
**SOM and Total C of a Rehabilitated Forest (Different Ages)**

| Age of Rehabilitated Forest Soil | Percentage (%) | |
|---|---|---|
| | SOM | Total C |
| Soil depth (0–20 cm) | | |
| 2008 (0 years old) | $3.485^{b}$ | $2.022^{b}$ |
| 2002 (6 years old) | $5.769^{a}$ | $3.346^{a}$ |
| 1996 (12 years old) | $3.762^{b}$ | $2.182^{b}$ |
| 1991 (17 years old) | $5.355^{a}$ | $3.106^{a}$ |
| Soil depth (20–40 cm) | | |
| 2008 (0 years old) | $3.370^{bc}$ | $1.955^{bc}$ |
| 2002 (6 years old) | $4.175^{ab}$ | $2.422^{b}$ |
| 1996 (12 years old) | $2.440^{c}$ | $1.415^{c}$ |
| 1991 (17 years old) | $5.492^{a}$ | $3.185^{a}$ |

*Note*: Means within column with different letters indicate significant difference in different ages by Duncan test at $p \leq 0.05$.

Soil depth did not significantly affect the total P and C/P ratios except for the 17-year-old rehabilitated forest, where the C/P ratio was greater at 0–20 cm than at 20–40 cm (Table 5). The following means comparison revealed that (Table 6): (1) total P in 0-, 6-, and 12-year-old rehabilitated forest irrespective of soil depth – no significant difference; (2) total P of 17-year-old rehabilitated forest irrespective of soil depth compared to those of 0-, 6-, and 12-year-old rehabilitated forest – significant difference; (3) C/P ratio irrespective of age of the rehabilitated forest at depth of 0–20 cm – no significant difference; (4) C/P ratio at depth of 20–40 cm of the 6-year-old rehabilitated forest compared to those of 0-, 12-, and 17-year-old rehabilitated forest – significant difference. Shukla[9] reported that accumulation of nutrients such as P increased with time.

**TABLE 5**
**Total P and C/P Ratios of a Rehabilitated Forest Soil (between Depths)**

| Age of Rehabilitated Forest Soil | Total P (%) | C/P Ratio |
|---|---|---|
| 2008 (0 years old) | | |
| 0–20 cm | $0.001^{a} \pm 0.0002$ | $4124.4^{a} \pm 1794.1$ |
| 20–40 cm | $0.002^{a} \pm 0.0002$ | $1598.8^{a} \pm 369.43$ |
| 2002 (6 years old) | | |
| 0–20 cm | $0.001^{a} \pm 0.0002$ | $20050^{a} \pm 14566$ |
| 20–40 cm | $0.001^{a} \pm 0.0001$ | $4674.1^{a} \pm 1301.3$ |
| 1996 (12 years old) | | |
| 0–20 cm | $0.002^{a} \pm 0.0004$ | $1354^{a} \pm 111.86$ |
| 20–40 cm | $0.001^{a} \pm 0.0002$ | $1369.1^{a} \pm 234.8$ |
| 1991 (17 years old) | | |
| 0–20 cm | $0.017^{a} \pm 0.0061$ | $507.89^{b} \pm 102.41$ |
| 20–40 cm | $0.005^{a} \pm 0.0012$ | $1543^{a} \pm 469.63$ |

*Note*: Means within column with different letters indicate significant difference between depths by independent t-test at $p \leq 0.05$.

**TABLE 6**
**Total P and C/P Ratios of a Rehabilitated Forest Soil (Different Ages)**

| Age of Rehabilitated Forest Soil | Total P (%) | C/P Ratio |
|---|---|---|
| Soil depth (0–20 cm) | | |
| 2008 (0 years old) | $0.0013^{b}$ | $4124^{ab}$ |
| 2002 (6 years old) | $0.0011^{b}$ | $20050^{a}$ |
| 1996 (12 years old) | $0.0021^{b}$ | $1354^{b}$ |
| 1991 (17 years old) | $0.0166^{a}$ | $508^{b}$ |
| Soil depth (20–40 cm) | | |
| 2008 (0 years old) | $0.0018^{b}$ | $1599^{b}$ |
| 2002 (6 years old) | $0.0011^{b}$ | $4674^{a}$ |
| 1996 (12 years old) | $0.0014^{b}$ | $1369^{b}$ |
| 1991 (17 years old) | $0.0052^{a}$ | $1543^{b}$ |

*Note*: Means within column with different letters indicate significant difference in different ages by Duncan test at $p \leq 0.05$.

In terms of depth, the statistical means comparison of the CEC showed no significant difference (Table 7). However, in terms of age, the CEC of the rehabilitated forest increased with increasing age (Table 8) and this observation could be partly related to the buildup of organic matter with time. This is because decomposition of organic matter affects soil CEC. The decomposition leads to production of humic substances, which are noted for increasing soil CEC because of their high carboxylic and phenolic functional groups whose exchange complexes have affinity for basic cations, such as Ca, Mg, K, and Na.

**TABLE 7**
**Soil CEC of a Rehabilitated Forest Soil (between Depths)**

| Age of Rehabilitated Forest Soil | CEC (cmol $kg^{-1}$) |
|---|---|
| 2008 (0 years old) | |
| 0–20 cm | $11.45^{a} \pm 1.789$ |
| 20–40 cm | $9.111^{a} \pm 2.069$ |
| 2002 (6 years old) | |
| 0–20 cm | $17.846^{a} \pm 2.290$ |
| 20–40 cm | $19.000^{a} \pm 2.522$ |
| 1996 (12 years old) | |
| 0–20 cm | $15.994^{a} \pm 2.423$ |
| 20–40 cm | $16.450^{a} \pm 2.176$ |
| 1991 (17 years old) | |
| 0–20 cm | $27.571^{a} \pm 2.265$ |
| 20–40 cm | $25.056^{a} \pm 1.780$ |

*Note*: Means within column with different letters indicate significant difference between depths by independent t-test at $p \leq 0.05$.

**TABLE 8**
**Soil CEC of a Rehabilitated Forest Soil (Different Ages)**

| Age of Rehabilitated Forest Soil | CEC (cmol $kg^{-1}$) |
|---|---|
| Soil depth (0–20 cm) | |
| 2008 (0 years old) | $11.45^{b}$ |
| 2002 (6 years old) | $17.846^{b}$ |
| 1996 (12 years old) | $15.994^{b}$ |
| 1991 (17 years old) | $27.571^{a}$ |
| Soil depth (20–40 cm) | |
| 2008 (0 years old) | $9.111^{c}$ |
| 2002 (6 years old) | $19.000^{b}$ |
| 1996 (12 years old) | $16.45^{b}$ |
| 1991 (17 years old) | $25.056^{a}$ |

*Note*: Means within column with different letters indicate significant difference in different ages by Duncan test at $p \leq 0.05$.

The higher pH (1 M KCl) at 20–40 cm compared to those at 0–20 cm (Table 2) of rehabilitated forest soil could be attributed to leaching of basic cations from 0–20 to 20–40 cm as the soil texture was loamy sand. However, this observation was not true for pH (water) because the KCl method indicates the potential acidity of the soil. The significant decrease in pH between different ages of the rehabilitated forest at the two soil depths suggests that age and depth of the forest affected the soil pH. This was because the rate of litter production of different ages and different plant species could be different. A similar finding was reported by Shukla[7]. Besides, the plants in the older forest might have a well-developed rooting system that led to higher root respiration. $CO_2$ produced might have dissolved in soil water to form weak acids that might have caused soil pH to decrease with age. Decaying of organic matter also produces strong acids and this might have also contributed to the decrease in the soil pH with increasing age of the rehabilitated forest.

With the exception of the 12-year-old (1996) rehabilitated forest whose SOM and total C were lower (Table 4) because of its lower soil pH (KCl) (Table 2) that might have retarded, for instance, production and decomposition of litter, the soil texture for all the ages of the rehabilitated forest was the same loamy sand. Thus, the general increase in SOM and total C with age regardless of depth could therefore be due to production and decomposition of litter, a process which leads to formation of humus. The age of a tree always correlates with litter production. Depending on species, older trees produce more litter that contributes to high litter accumulation and high buildup of organic matter. This observation is consistent with that reported by Rajeswari et al.[10], who reported that infiltration rate of forest soil was affected by high organic matter content in different ages of a rehabilitated forest.

The high total C at 0–20 cm suggests that higher decomposition of biomass may have occurred through the decomposition of plant and animal residues, root exudates, living and dead microorganisms, and soil biota. The accumulation may also have been possible. Rate of organic matter decomposition partly depends on soil pH and level of nutrients. The acidic nature of the soil may have limited the abundance of soil microbes and their ability to decompose biomass to release more C. Thus, soil with high acidity regardless of depth usually tends to have low organic matter. The 0-year-old rehabilitated forest (0–20 cm) showed the least percentage of organic matter, probably due to the compact nature of the soil as the roots of young trees in the plot were yet to establish well as compared to the older trees. This leads to poor P absorption by plant roots and also limits soil microbial activities.

The fact that the percentage of total P regardless of depth increased while C/P ratio decreased with age suggests that there was P available in the soil with increasing age of the rehabilitated forest. Availability of P in the soil correlates with organic matter[11]. Soils with high organic matter contain considerable amounts of organic P, which are mineralized to provide available P for plant growth. According to Havlin et al.[11], soil P mineralization increases with increasing organic P content, while the amount of inorganic P immobilization is inversely associated with soil organic P in such a way that, as the ratio of soil organic C/P ratio increases, P immobilization increases. Even though soil aeration also affects C/P ratio, in this study this factor may not have played a significant role because the soil texture indirectly affects the soil aeration and for that matter C/P ratio. Since the role of P is essential for the general health and vigor of all plants, the adequate supply of P to the plant would induce better plant growth.

## CONCLUSION

Regardless of soil depth, pH of the rehabilitated forest decreased with time. However, SOM, total C, and total P increased with age of the rehabilitated forest irrespective of depth, except for C/P ratio, which decreased with time at the depth of 0–20 cm only. The rehabilitated forest has shown signs of being a C and P sink, as C and P accumulation increased with time.

## ACKNOWLEDGMENT

The authors acknowledge the financial support of Universiti Putra Malaysia and Mitsubishi Corporation.
